# Potential Mechanisms of *AtNPR1* Mediated Resistance against Huanglongbing (HLB) in *Citrus*

**DOI:** 10.3390/ijms21062009

**Published:** 2020-03-16

**Authors:** Wenming Qiu, Juliana Soares, Zhiqian Pang, Yixiao Huang, Zhonghai Sun, Nian Wang, Jude Grosser, Manjul Dutt

**Affiliations:** 1 Institute of Fruit and Tea, Hubei Academy of Agricultural Sciences, Wuhan 430064, China; qiuwm1984@163.com (W.Q.); hbfruit@126.com (Z.S.); 2 Citrus Research and Education Center, University of Florida, Lake Alfred, FL 33850, USA; jmoreirasoares@ufl.edu (J.S.); pangzq@ufl.edu (Z.P.); yixiaohuang@ufl.edu (Y.H.); nianwang@ufl.edu (N.W.); jgrosser@ufl.edu (J.G.)

**Keywords:** *Citrus sinensis*, disease resistance, HLB, innate immunity, RNAseq

## Abstract

Huanglongbing (HLB), a bacterial disease caused by *Candidatus* Liberibacter asiaticus (*C*Las), is a major threat to the citrus industry. In a previous study conducted by our laboratory, several citrus transgenic trees expressing the *Arabidopsis thaliana NPR1* (*AtNPR1*) gene remained HLB-free when grown in a field site under high HLB disease pressure. To determine the molecular mechanisms behind *AtNPR1-*mediated tolerance to HLB, a transcriptome analysis was performed using *AtNPR1* overexpressing transgenic trees and non-transgenic trees as control, from which we identified 57 differentially expressed genes (DEGs). Data mining revealed the enhanced transcription of genes encoding pathogen-associated molecular patterns (PAMPs), transcription factors, leucine-rich repeat receptor kinases (LRR-RKs), and putative ankyrin repeat-containing proteins. These proteins were highly upregulated in the *AtNPR1* transgenic line compared to the control plant. Furthermore, analysis of protein–protein interactions indicated that AtNPR1 interacts with CsNPR3 and CsTGA5 in the nucleus. Our results suggest that *AtNPR1* positively regulates the innate defense mechanisms in citrus thereby boosting resistance and effectively protecting the plant against HLB.

## 1. Introduction

Huanglongbing (HLB), also known as citrus greening, is caused by the phloem-limited pathogenic bacterium *Candidatus* Liberibacter asiaticus (*C*Las) and is the most destructive disease in citrus plants [1]. *C*Las infects most *Citrus* cultivars, including sweet orange, mandarin, lemon, and grapefruit, and causes severe disease symptoms, with severely infected trees experiencing a reduction in fruit yield and quality, leading to the plant’s eventual decline and death. Tolerance to HLB has been reported in citron and in some trifoliate oranges and their hybrids [2]. To date, there is no definitive cure for HLB; however, to prevent the further spread of disease, methods such as insect vector control, tree health management, and the destruction of infected plants are necessary [3,4].

Several treatments have been used to control the spread of HLB in citrus trees, including antibiotic and antimicrobial treatments [5,6], heat treatment [7], and trunk injection of plant defense activators [8]. Moreover, to manage the disease, several plant defense inducers have been sprayed on HLB-infected field trees [9]. The premise behind these studies is that induced resistance, either in a local manner or spread systemically throughout the trees, could confer long-lasting protection against HLB by activating the salicylic acid (SA) signaling or systemic acquired resistance (SAR) pathways [10]. The *NONEXPRESSOR OF PATHOGENESIS RELATED GENE 1* (*NPR1*) is a crucial regulator of SAR and plays an essential role in SAR activation [11,12]. In response to pathogen infection, an accumulation of SA is observed, and NPR1 oligomers present in the cytoplasm are reduced to their monomeric form, after which they move to the nucleus of the plant cells. In the nucleus, NPR1 protein binds to TGA transcription factors to induce *PATHOGENESIS RELATED 1* (*PR1*) gene expression [13,14,15,16,17]. The three TGAs (TGA2, TGA5, and TGA6) function redundantly to positively regulate SA-induced immune responses [17]. In *Arabidopsis*, *NPR1* paralogs *NPR3/NPR4* were identified as SA receptors. The NPR1 and NPR3/NPR4 proteins function independently and play opposite roles in SA-induced pathogen resistance [18,19].

As a key regulator of SAR, *NPR1* confers long-lasting broad-spectrum resistance in plants. In rice, the expression of *Arabidopsis NPR1* (*AtNPR1*) transgene not only enhances resistance to the bacterial pathogen *Xanthomonas oryzae* pv. *oryzae* but also induces a lesion-mimic/cell death phenotype [20,21]. *NPR1*, as well as other genes in its pathway in crops such as wheat [22], tobacco [23], soybean [24], and sweet sagewort [25], were considered to be effective candidates for engineering transgene-mediated disease resistance. In addition to disease resistance, the NPR1-dependent SA signaling pathway plays a role in abiotic stress, as it modulates reactive oxygen species, proline, and redox states during salt and oxidative stresses [26], drought stress [27], and cold acclimation [28].

The overexpression of *AtNPR1* or its orthologs resulted in enhanced resistance to biotic and abiotic stress in several fruit and vegetable crops such as grape, carrot, tomato, apple, citrus, tobacco, and strawberry [29]. In citrus, overexpressing *AtNPR1* in ‘Duncan’ grapefruit and ‘Hamlin’ sweet orange conferred increased resistance to citrus canker [30]. ‘Hamlin’ and ‘Valencia’ sweet orange transgenic lines ectopically expressing *AtNPR1* under the control of the cauliflower mosaic virus (CaMV) 35S promoter or the *Arabidopsis thaliana SUCROSE SYNTHASE 2* promoter (*AtSUC2*, a phloem specific promoter) has remained HLB-free in a site of high disease pressure [31]. In that study, several plant defense related genes were highly upregulated, however, a detailed study to elucidate the underlying mechanisms behind HLB resistance was not performed. In this study, an in-depth analysis, including a study of the transcriptome of the HLB resistant transgenic line, was conducted. 

## 2. Results

### 2.1. Transcriptome Sequencing and Differentially Expressed Genes (DEGs) Identification

To investigate the transcriptional responses associated with *AtNPR1* overexpression in ‘Valencia’ sweet orange plants, we performed RNA sequencing (RNAseq) analyses on a selected NPR overexpressing line and a non-transgenic control plant. Our previous studies showed that the NPR1-2 transgenic line consistently expressed the highest levels of the transgene, as such, this line was the transgenic line of choice and ‘Valencia’ (Val) the non-transgenic control. Leaf samples from three independent replicates were collected, and total RNA for Illumina RNAseq was extracted, from which six cDNA libraries were constructed. 

**Figure 1:**
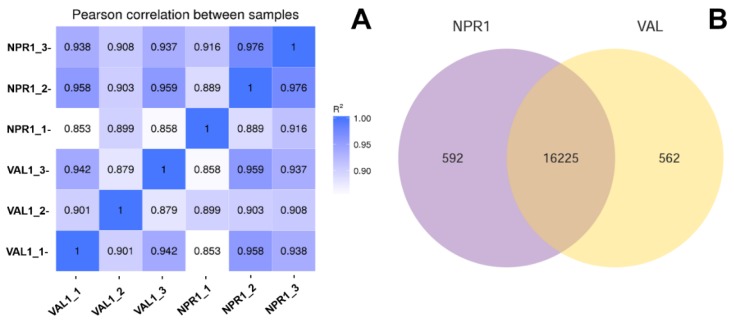
RNAseq analysis (**A**) Correlation between RNA-Seq samples. NPR1_1/2/3 represent three replicates of the NPR1 overexpressing line (NPR1-2), Val_1/2/3 represent three replicates of the control non-transgenic ‘Valencia’ line, heat maps of the correlation coefficient between samples, R^2^ means the square of the Pearson coefficient; (**B**) Venn diagram of expressed genes in transgenic NPR1 overexpressing line (NPR1-2) and control non-transgenic ‘Valencia’ line. FPKM > 1 is the expression threshold, NPR1 and Val represent the *AtNPR1*-transgenic line (NPR1-2) and non-transgenic ‘Valencia’ line, respectively.

The raw data were filtered and mapped as shown in Table 1. Results showed 373.65 million clean reads with a total of 47 billion guanine (G) base numbers. The percentage of high-quality reads (a quality score higher than 30 (Q30) indicates that the base call accuracy of each read is more than 99.9%) in each library was more than 92%. Afterwards, the clean reads were mapped to the reference genome using the TopHat2 software, showing at least 76% reads of each sample mapped to the reference genome. These results indicated absence of contamination and that the reference genome was appropriately chosen, since more than 73% reads could be uniquely mapped (Table 1). In this study, R^2^ was larger than 0.85 for both the tested samples (Figure 1A), demonstrating the experiment’s reliability and its usefulness in revealing differences in gene expression between samples.

**Figure 2:**
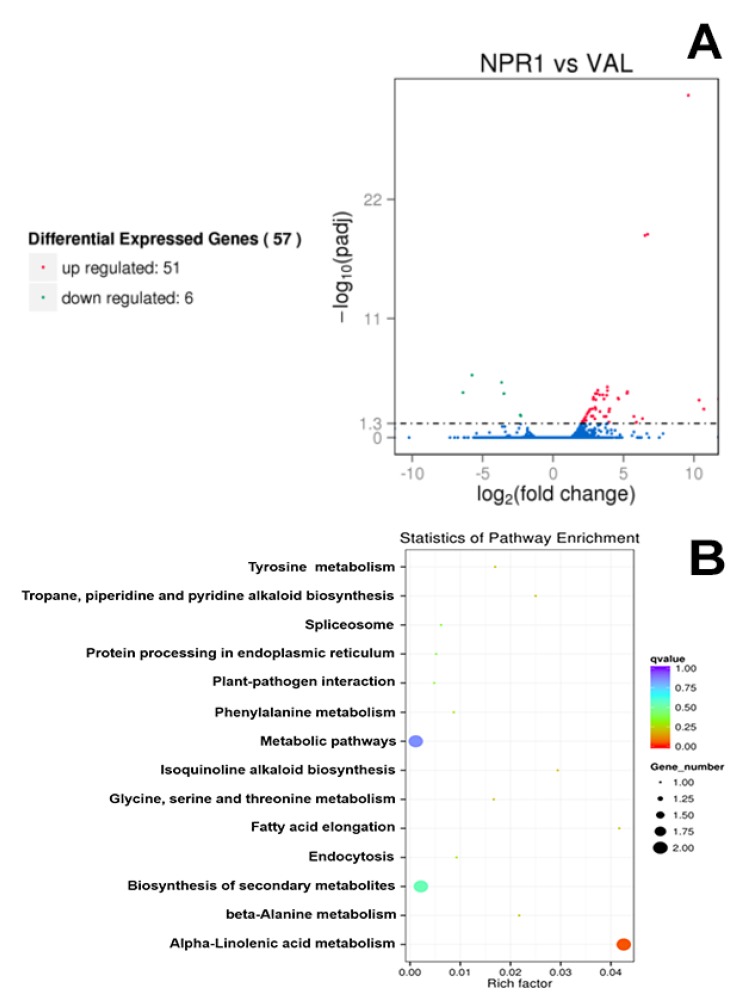
Analysis of the differentially expressed genes (DEGs). (**A**) DEGs between the transgenic NPR1 overexpressing line (NPR1-2) and control non-transgenic ‘Valencia’ line; (**B**) Kyoto Encyclopedia of Genes and Genomes (KEGG) enrichment scatter plot of DEGs. The y-axis shows the name of the pathway and the x-axis shows the Rich factor. Dot size represents the number of different genes and the color indicates the q-value.

In total, 16,817 and 16,787 expressing genes were identified in the transgenic and control lines, respectively. Among those, 592 genes were specific to the transgenic line, whereas 562 genes were specifically expressing in the control Val as demonstrated in the Venn diagram (Figure 1B). The differentially expressed genes (DEGs) between NPR1-2 and Val were screened using the DESeq software with padj < 0.05, from which 57 DEGs were identified, of which there were 51 upregulated genes and six downregulated genes (Figure 2A). The DEGs were annotated using the sequence of the best hit gene in the reference genome. The functional description determined by BLASTx alignment indicated the presence of 40 upregulated DEGs and five downregulated DEGs which represent functional genes, whereas 11 DEGs were determined to be nonfunctional. The upregulated DEGs were composed of at least five genes encoding ankyrin repeat-containing proteins, four coding for mechanosensitive ion channel protein including a *CYCLIC NUCLEOTIDE-GATED ION CHANNEL 1* (*CNGC1*) and four *LEUCINE-RICH REPEAT RECEPTOR KINASES* (LRR-RKs). In addition, putative pathogen-associated molecular patterns (PAMP) transcription factors such as *MYB4*, *NAC68*, and *WRKY51/70* were also upregulated. Among the five downregulated DEGs, three belong to the MADS-box family gene (including *AGL8* and *SEP2*), one is 3-*KETOACYL-COA SYNTHASE 11*, and one is *GERANIOL 8-HYDROXYLASE* (Table 2).

**Figure 3:**
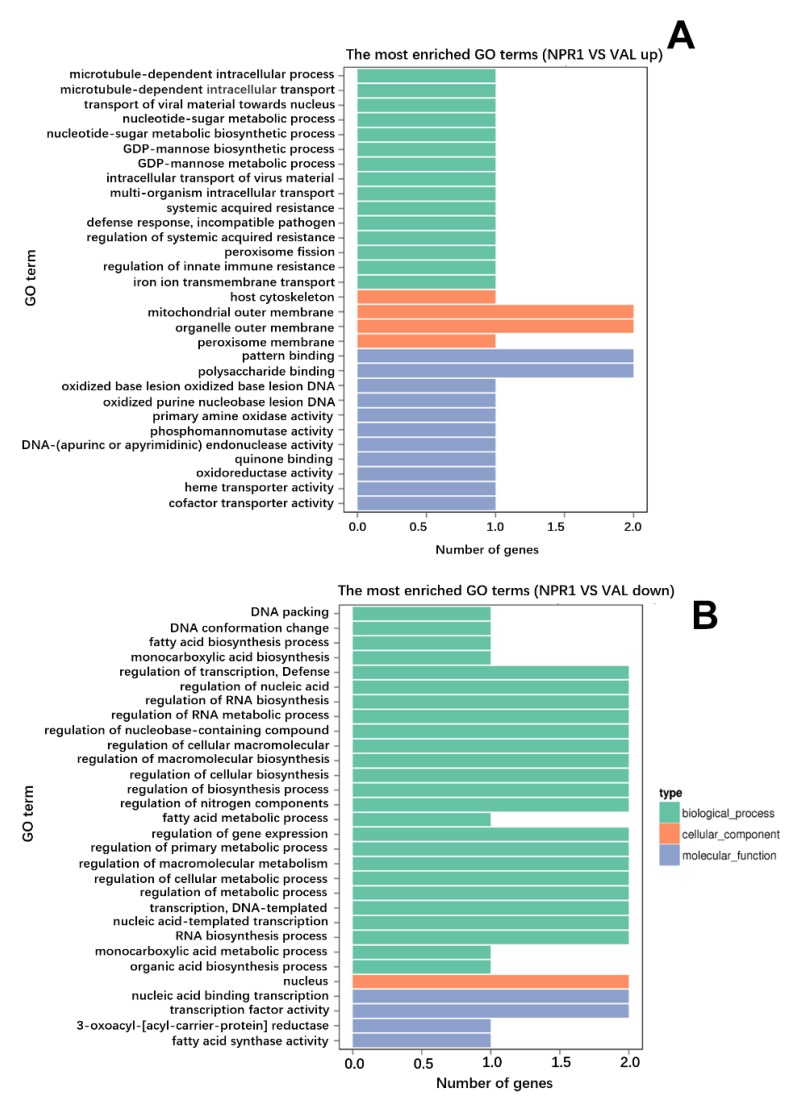
Gene ontology (GO) term enrichment analysis. (**A**,**B**) The GO enrichment terms of (A) upregulated (B) and downregulated DEGs. In the x-axis the number of DEGs is represented, and in the y-axis the GO terms enriched. Different colors are used to distinct biological process, cellular components, and molecular function.

To reveal the differences in metabolic pathways, DEGs were analyzed using the Kyoto Encyclopedia of Genes and Genomes (KEGG) pathway enrichment analysis, from which a total of 17 DEGs were annotated in 14 pathway terms. However, only the pathway involved in alpha-linolenic acid metabolism was significantly enriched at the corrected *p*-value threshold of < 0.05 (Figure 2B). Gene ontology (GO) enrichment analysis showed the functional classification of 28 GO terms, but no enriched GO terms were obtained using a corrected *p*-value threshold of < 0.05 (Figure 3).

### 2.2. Expression of DEGs Candidate in RNAseq Correlates with qRT-PCR

**Figure 4:**
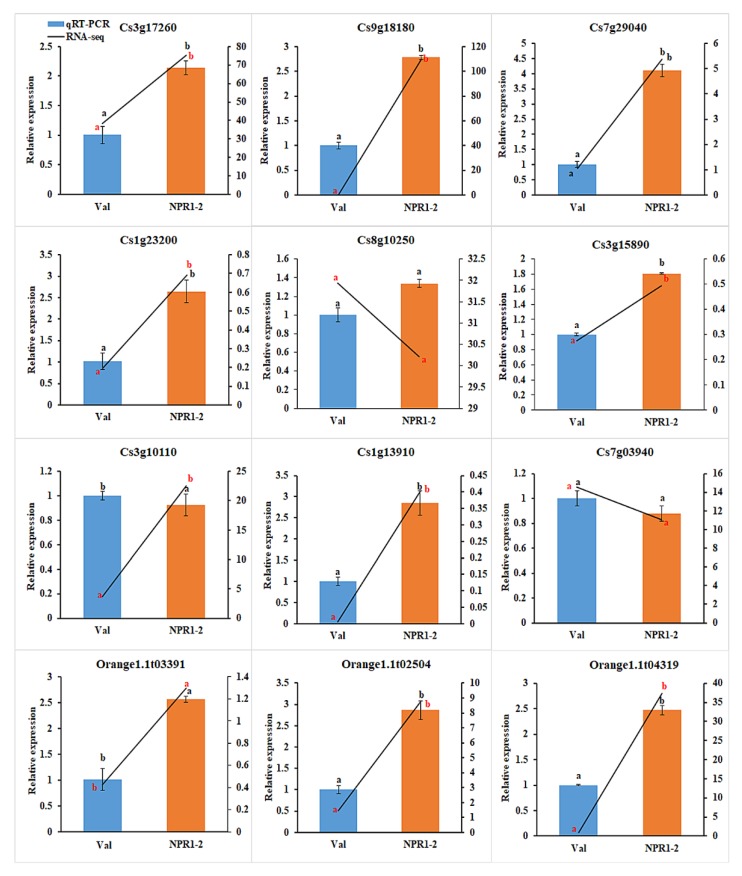
qRT-PCR verification for expression pattern of selected DEGs. The expression levels of DEG candidates in NPR1-2 transgenic line compared to Val control determined by qRT-PCR (2^-ΔΔCt^). Different letters (a, b) represent a significant difference at *p* ≤ 0.05 using Duncan’s Multiple Range Test and error bars represent SE (*n* = 3).

To verify the accuracy of the RNAseq results, 12 DEGs were selected for quantitative PCR (qRT-PCR) validation (Supplementary Tables S1 and S2). The qRT-PCR and RNAseq expression levels of selected DEGs were calculated using the 2^-ΔΔCt^ method and the fragments per kilobase of exon per million reads (FPKM) value, respectively. The results indicated that 11 DEGs, such as the *CYCLIC NUCLEOTIDE-GATED ION CHANNEL 1*, *HEAT SHOCK COGNATE 70 KDA PROTEIN*, *LEUCINE RICH REPEAT* (*LRR*) *RECEPTOR-LIKE SERINE/THREONINE-PROTEIN KINASE*, *WALL-ASSOCIATED RECEPTOR KINASE-LIKE 10*, and the ankyrin repeat-containing protein, showed consistent expression tendencies between both techniques, indicating the reliability of the RNAseq results for gene quantification analysis (Figure 4).

**Figure 5:**
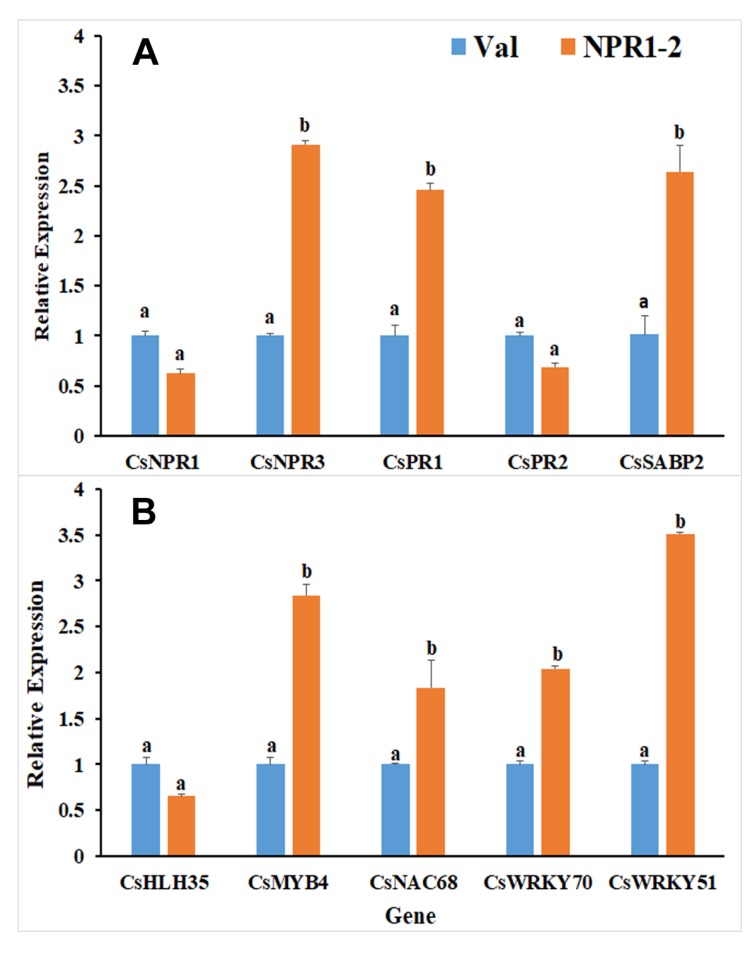
qRT-PCR for RNAseq data validation of genes involved with plant defense responses. Comparison between NPR1-2 transgenic and non-transgenic plants of genes involved in the (**A**) NPR1-SA dependent pathway, including CsNPR1, CsNPR3, and CsSABP2 along with pathogenesis related proteins, CsPR1 and CsPR2; and (**B**) transcription factors. Different letters (a, b) represent a significant difference at *p* ≤ 0.05 using Duncan’s Multiple Range Test and error bars represent SE (*n* = 3).

To get more insights regarding the defense-related pathways in the transgenic line, the expression levels of 10 other DEGs were quantified using qRT-PCR, wherein three of these genes are involved in the salicylic acid (SA) signaling pathway, two are *PATHOGENESIS-RELATED* genes, and five are transcription factors (TF) that function downstream of the NPR1 protein in the pathway. In comparison to the non-transgenic control (Val), *Citrus sinensis NPR3* (*CsNPR3*), *CsPR1*, and *CsSABP2* genes were upregulated in the NPR1-2 line whereas *CsPR2* was significantly downregulated (Figure 5). Among the five TFs, only bHLH35 had reduced expression in line NPR1-2, whereas *MYB4*, *NAC68*, *WRKY70*, and *WRKY51* were upregulated.

### 2.3. AtNPR1 Interacts with Citrus Homolog Proteins

The upregulation of several ankyrin repeat-containing proteins and defense related genes in the NPR1-2 transgenic line as observed in our RNAseq data prompted us to investigate whether AtNPR1 could interact with citrus homolog proteins. As we are focusing on the NPR1-dependent SA signaling pathway, we chose components of the pathway already known from *Arabidopsis* model plant research such as the NPR3 and TGA5. The citrus CsNPR3 homolog protein share 60% of similarity with the ankyrin-repeat domain proteins induced in our RNA-seq. The AtTGA transcription factors interact with AtNPR1 in the nucleus to physically bind to the *PR1* promoter [32].

**Figure 6:**
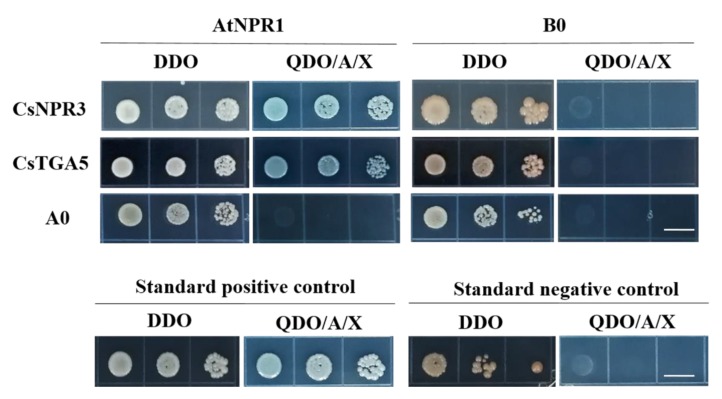
Yeast-two-hybrid (Y2H) interaction assays of AtNPR1 with CsNPR3 and CsTGA5. DDO (double dropout medium): SD/-Trp and -Leu, QDO (quadruple dropout medium)/X/A: SD/-Leu/-Trp/-Ade/-His + X-α-Gal and Aureobasidin A. The empty BD (B0) and AD (A0) vectors were used as negative controls. Standard positive control (pGBKT7-53 and pGADT7-T; Clontech) and standard negative control (pGBKT7-Lam and pGADT7-T) were included. The scale bar at the bottom of the figure denotes 1 cm length.

In this analysis, AtNPR1 was used as prey and CsNPR3 and CsTGA5 were used as baits. We detected positive interactions between the AtNPR1 protein and both citrus homologs using yeast two-hybrid system (Y2H) assays (Figure 6).

**Figure 7:**
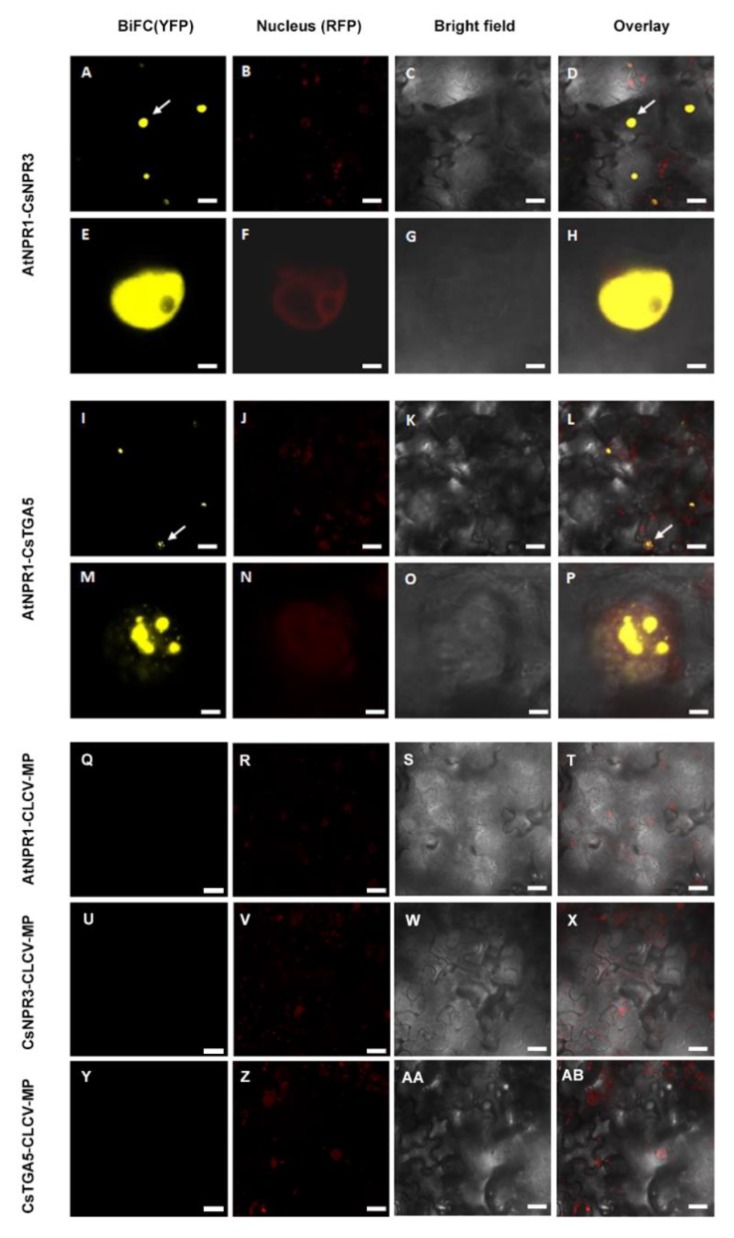
Confocal micrographs showing results of bimolecular fluorescence complementation assays (BiFC) assays to determine interactions between AtNPR1 with CsNPR3 and CsTGA5 proteins. (**A**–AB) show micrographs of YFP (BiFC), nuclear marker (Nucleus), transmission white light (Bright field) and the resultant overlay, respectively. The upper set of panels are showing positive interaction and the bottom set of pictures are showing the negative controls. (**A**–**H**) show the AtNPR1 and CsNPR3 positive interaction. (**E**–**H**) is a zoom up containing nuclei details. In (**I**–**P**) the positive interaction between AtNPR1 and CsTGA5 is demonstrated and (**M**–**P**) is a zoom up showing nuclei. White arrows indicate nuclear interaction. (**Q**–**AB**) pictures show the negative controls. All the constructs were transiently co-expressed in *Nicotiana benthamiana* RFP-H-2B plants. The scale bars represent 35 µM in length and for the magnified images each bar represent 5 µM in length.

To confirm the positive interactions, bimolecular fluorescence complementation assays (BiFC) were performed, and similar to the Y2H assay, AtNPR1 was observed to be capable of interacting with both the CsNPR3 and CsTGA5 proteins. The yellow fluorescence indicating a positive interaction, was observed to have nuclear localization (Figure 7).

## 3. Discussion

To our knowledge, this study is the first report on the transcriptional analysis of a citrus transgenic plant that shows tolerance to HLB. Transcriptomic, proteomic, and metabolomic analyses were performed to further examine citrus genomics, from which candidate genes/proteins/metabolites were identified; however, the majority of previous studies compared samples infected with *C*Las to those without infection, or compared tolerant and susceptible citrus cultivars [33,34,35,36,37,38,39]. 

### 3.1. The Transcriptional Change in NPR1-2 Transgenic Line

Among the DEGs identified in this study, three isoforms of *CYCLIC NUCLEOTIDE-GATED ION CHANNEL* (*CNGC*) were upregulated in the NPR1-2 transgenic line. *CNGCs* are ion channels regulated by cytosolic signaling molecules (i.e., cyclic nucleotides, calmodulin, and Ca^2+^). Upon mechanical wounding or insect injury, a quick increase in Ca^2+^ influx takes place, activating Ca^2+^/CaM-dependent phosphorylation, resulting in a rapid burst of jasmonic acid (JA) and the activation of plant defense against herbivory [40]. In *Arabidopsis*, *AtCNGC1* partly contributes to Ca^2+^ uptake into plants (along with other channels), affecting the primary root growth of seedlings [41]. *AtCNGC2* and *AtCNGC4* are essential for the activation of PAMP-triggered immunity upon pathogen infection of *Arabidopsis* plants [13]. Together, these proteins are assembled into a functional calcium channel, which becomes phosphorylated and activated by the effector kinase *BOTRYTIS-INDUCED KINASE1* (*BIK1*) of the pattern-recognition receptor complex [42]. *AtCNGC11* and *AtCNGC12* induce a hypersensitive response (HR) and act as positive mediators of multiple pathogen resistant responses involved in SA signaling pathways and PR protein accumulation [43,44]. 

Other genes upregulated in the transgenic line were the *LRR RECEPTOR-LIKE SERINE/THREONINE-PROTEIN KINASE* and *WALL-ASSOCIATED RECEPTOR KINASE-LIKE*. The receptor-like kinases (*RLKs*) are plant extracellular receptors involved in pathogen recognition and the subsequent activation of plant defense responses [45,46]. The *Arabidopsis LRR RECEPTOR-LIKE PROTEIN KINASES* modulates brassinosteroid, abscisic acid, SA, and Ca2^+^ signaling, and positively regulates diverse responses against stresses such as pathogen infection, cold, salt, and aphid attacks [47,48]. In maize, a *WALL-ASSOCIATED RECEPTOR-LIKE KINASE* was found to confer quantitative field resistance against most Northern corn leaf blights [46]. 

*MYB4*, *NAC68*, *WRKY70*, and *WRKY51* are the TFs found upregulated in NPR1-2. The *WRKY* family of TFs are exclusively found in plants and play an essential role in plant signaling, modulating several plant responses to biotic and abiotic stimuli. Both of the *WRKY* TFs upregulated in NPR1-2, namely, *WRKY70* and *WRKY51*, are known to be involved in the cross-talk between the SA and the jasmonic acid (JA)/ethylene (ET) signaling pathways, and can regulate plant defense either positively or negatively [49,50,51]. *WRKY70* activation of genes related to the SA signaling pathway and the simultaneous repression of JA pathway responses is partly coordinated by NPR1-dependent mechanisms; as such, *WRKY70* is capable of fine tuning SA- and JA-dependent defenses [51]. Similarly, *WRKY51* is also involved with repression of the JA signaling pathway; however, this repression is mediated by the reduction of oleic acid (18:1) levels. In *Arabidopsis*, an upregulation of SA-mediated responses is induced in low-18:1 background, leading to an increase in resistance to biotrophic but not to necrotrophic pathogens [50].

Previously, we demonstrated an induction of *WRKY70* in the transgenic lines ectopically expressing *AtNPR1* transgene either under the constitutive 35S promoter or the phloem-specific *AtSUC2* promoter, which correlated with our RNAseq findings and the notion that NPR1 modulates WRKY70 to trigger plant defense [31]. Thus, it is reasonable to assume that an increase in expression of the DEGs, CNGCs, receptor-like kinases, and the plant defense response TFs could contribute to the enhancement of tolerance to HLB in NPR1-2 transgenic plants.

### 3.2. AtNPR1 Interacted with NPR Orthologs Proteins

The NPR1-dependent pathway involves activation through a complex signaling network and requires an accumulation of the SA phytohormone and different protein–protein interactions. The *Arabidopsis* isoforms of NPR, NPR1, NPR3/4, and TGA TFs need to interact before defense can be triggered [18,52,53]. Interestingly, AtNPR1 was found to be capable of interacting with the citrus isoforms of NPR3 and TGA5; therefore, the AtNPR1 transgene in the NPR1-2 line may be recruiting the citrus NPR orthologs to regulate the innate defense mechanisms against HLB.

AtNPR1 overexpressing transgenic trees showed enhanced transcription of genes encoding for pathogen-associated molecular patterns (PAMP), transcription factors, leucine-rich repeat receptor kinases (LRR-RKs), and putative ankyrin repeat-containing proteins. Furthermore, analysis of protein–protein interactions demonstrated that AtNPR1 could interact with CsNPR3 and CsTGA5 in the nucleus indicating that the involvement of AtNPR1 transgene in the citrus SA-pathway could possibly regulate innate defense mechanisms boosting resistance and protecting the plant against HLB.

## 4. Materials and Methods

### 4.1. Plant Materials

‘Valencia’ (*Citrus sinensis* Osbeck) sweet orange lines overexpressing the *Arabidopsis thaliana NPR1* (*AtNPR1*; AT1G64280) cDNA under the control of a constitutive CaMV 35S promoter have been previously identified [31]. A selected transgenic and non-transgenic line were clonally propagated onto Carrizo citrange (*Citrus sinensis* (*L.*) *Osbeck* × *Poncirus trifoliata* (L.) Raf.) rootstock. Leaf samples from one-year-old trees maintained in greenhouse located at the University of Florida’s Citrus Research and Education Center (Lake Alfred, FL, USA) were used for RNA extraction.

### 4.2. RNA Extraction and cDNA Synthesis

RNA was extracted using TRIzol^®^ [54], following the manufacturer’s protocol. RNA concentration was determined using a NanoDrop™ 1000 Spectrophotometer (Thermo Fisher Scientific, Franklin, MA, USA). The purity and integrity of the RNA were analyzed using electrophoresis on a 1.0% agarose gel, and then examined using the Agilent 2100 Bioanalyzer (Agilent Technologies, Santa Clara, CA, USA). High quality RNA samples with RNA integrity number (RIN) value > 6.5 were used for cDNA synthesis and RNA-sequencing. Single-strand cDNA was synthesized using the RevertAid First Strand cDNA Synthesis Kit (Thermo Fisher Scientific, Franklin, MA, USA).

### 4.3. RNAseq Libraries Construction and Sequencing

Approximately 3 μg of RNA was used for sequencing library construction using the NEBNext^®^ Ultra™ RNA Library Prep Kit for Illumina^®^ (New England Biolabs, Ipswich, MA, USA) following the manufacturer’s instructions. Briefly, mRNA was enriched using oligo (dT) beads and then fragmented randomly, after which first-strand cDNA was synthesized using random hexamers and M-MuLV reverse transcriptase. Second strand cDNA synthesis was subsequently performed using DNA Polymerase I and RNase H. After adenylation of the 3′ ends of DNA fragments, NEBNext^®^ adaptors were ligated to prepare for hybridization. cDNA fragments of 150-200 bp in length were purified using the AMPure XP system (Beckman Coulter, Brea, CA, USA). Afterwards, 3μL USER Enzyme (New England Biolabs, Ipswich, MA, USA) was added to size-selected, adaptor-ligated cDNA, and was incubated at 37 °C for 15 min, followed by 5 min at 95 °C to facilitate hybridization. PCR was performed using Phusion High-Fidelity DNA polymerase (New England Biolabs, Ipswich, MA, USA), the products were purified using the AMPure XP purification system (Beckman Coulter, Brea, CA, USA), and library quality was assessed on the Agilent 2100 Bioanalyzer. The library was sequenced using an Illumina^®^ HiSeq 2000 platform, and 125 bp/150 bp paired-end reads were generated. Library construction and sequencing were performed by Novogene Corporation (Novogene, Sacramento, CA, USA). For each line, three technical replicates were created. The raw data was deposited into the National Center for Biotechnology Information Sequence Read Archive (https://www.ncbi.nlm.nih.gov/sra, accessed on 11/2/2020) with a SRA accession number: PRJNA602381.

### 4.4. Data Mapping and Differentially Expressed Genes Identification

Raw reads that were in FASTQ format were firstly processed using in-house Perl scripts. Clean reads were obtained by removing reads containing adapters or reads of low quality; these clean reads were used for downstream analyses. TopHat (v2.0.12) software (Johns Hopkins University, Baltimore, MD, USA) was chosen to map the clean reads to the sweet orange genome (http://citrus.hzau.edu.cn/orange/, accessed on 27/2/2017), with mismatch parameter set to two and other parameters set to default. Gene expression level was calculated by counting the reads that map to genes or exons. The FPKM (Fragments Per Kilobase of transcript sequence per Millions base pairs sequenced) value was used to normalize the read counts to estimate gene expression levels, as this takes into account the effects of both sequencing depth and gene length on the counting of fragments. In this study, the final FPKM was calculated as the mean value of the three technical replicates. DESeq software was used to analyze the differentially expressed genes (DEGs) using a negative binomial distribution *p*-value estimation model, with the differentially expressed gene screening standard set to padj < 0.05 [55]. To annotate the DEGs, BLASTx alignment (E-value < 10^-5^) was performed based on the UniProtKB/Swiss-Prot database (https://www.uniprot.org/blast/, accessed on 27/2/2017). 

### 4.5. GO and KEGG Pathway Analysis

Gene Ontology (GO) enrichment analysis was performed using GOseq. A GO enrichment bar chart was used to illustrate the DEG-enriched GO terms as well as the DEG counts for each GO term. Overrepresented *p*-values in the hypergenometric test were used to identify significantly enriched GO terms with corrected *p*-values < 0.05 [56]. The Kyoto Encyclopedia of Genes and Genomes (KEGG) pathway enrichment analysis was used to identify significantly enriched metabolic pathways or signal transduction pathways associated with DEGs when compared to the whole genome background. A scatter diagram was used to display KEGG enrichment analysis results. The KEGG enrichment degree was measured based on the rich factor, Q value, and gene counts enriched in this pathway. Pathways with corrected *p*-values < 0.05 were determined to be significantly enriched in DEGs. As the number of enriched pathways counts was less than 20, all of them were plotted.

### 4.6. Quantitative Real-Time RT-PCR (qRT-PCR) Analysis

Selected gene-specific primers (Supplementary Table S1) were designed using the online real-time PCR tool provided by Integrated DNA Technologies, Inc (Integrated DNA Technologies, Coralville, IA, USA; https://www.idtdna.com, accessed on 26/9/2018). The final volume of reaction mixture containing 1× PowerUp™ SYBR^®^ Green Master Mix (Thermo Fisher Scientific, Franklin, MA, USA), 50 ng cDNA, and 500 nM forward and reverse primers were 10 µL, and three replicates for each reaction were prepared. qRT-PCR was performed in a StepOnePlus™ Real-Time PCR System (Thermo Fisher Scientific, Franklin, MA, USA). Cycling conditions were set as follows: 95 °C denaturation for 15 s, followed by 40 cycles of 95 °C denaturation for 15 s and 60 °C annealing and extension for 1 min. To test for PCR specificity, the melting curve was generated by gradually increasing the temperature to 95 °C. The citrus β-actin housekeeping gene was used as the reference gene [57]. Relative gene expression was calculated using the 2^-ΔΔCt^ method described previously [58].

### 4.7. Vector Construction Using Gateway Technology

Q5^®^ High-Fidelity 2× Master Mix (New England Biolabs, Ipswich, MA, USA) was used for all gene amplification reactions. The primers used and gene accession numbers are listed in Supplementary Tables S2 and S3. PCR products were purified using the QIAquick Gel Extraction Kit (QIAGEN, Germantown, MD, USA) and cloned into the Gateway pDONR211 entry clone using the BP recombination reaction (Thermo Fisher Scientific, Franklin, MA, USA). Clones were transformed into competent DH5α *E. coli* cells using the heat-shock method. After validation through Sanger sequencing, the correct clones were recombined into pSITE-c/nYFP-C1/N1 destination vectors using Gateway^®^ LR Clonase^®^ II enzyme mix (Thermo Fisher Scientific, Franklin, MA, USA) according to the manufacturer’s protocol. Positive colonies were selected using LB plates supplemented with spectinomycin; these were confirmed to have the plasmid through PCR, and plasmid DNA was extracted using the GeneJET Plasmid Miniprep Kit (Thermo Fisher Scientific, Franklin, MA, USA). The destination clones were introduced into *Agrobacterium tumefaciens* strain EHA105 by freeze-thaw method [59]. Positive EHA105 clones were validated by PCR.

### 4.8. Bimolecular Fluorescence Complementation Assays

Bimolecular fluorescence complementation (BiFC) assays using a split YFP system were conducted according to previously described protocol [60]. Vectors were generated with Gateway technology as described above. As negative control, *Cabbage leaf curl virus* (*CLCV*) movement protein (CLCV-MP) were used. All of the BiFC assays were performed in *Nicotiana benthamiana* transgenic plants that constitutively express RFP fused to histone 2B (*N. benthamiana* RFP-H2B), generated by Martin, et al. [61]. The CLCV-MP constructs and *N. benthamiana* RFP-H2B seeds were kindly donated by Dr. Amit Levy. Transient expression of fusion proteins in *N. benthamiana* was performed according the methods described by Sparkes et al. [62] with minor modifications. A single colony of *Agrobacterium* strain EHA105 containing each destination clone was grown in Luria–Bertani broth containing rifampicin (50 mg/L) and spectinomycin (100 mg/L) incubated at 28 °C overnight. The culture was centrifuged and re-suspended in infiltration buffer (10 mM 2-(N-morpholino) ethanesulfonic acid (MES), pH 5.85; 10 mM MgCl_2_) containing 200 µm acetosyringone. The infiltration buffer was kept at room temperature for 3–4 h and infiltrated into the leaves using a 1 mL needleless syringe. The plants were evaluated and photographed 3 days after infiltration using a confocal microscope (Leica Microsystems Inc., Buffalo Grove, IL, USA).

### 4.9. Yeast Two-Hybrid Assays

Yeast vectors pGBKT7 (GAL4 DNA-binding domain, BD), pGADT7 (GAL4 activation domain, AD), and the yeast strain Y2HGold (Clontech, Mountain View, CA, USA) were used in the yeast two-hybrid assays. To test the interaction between AtNPR1 and the candidate target proteins (CsNPR3 and CsTGA5), cDNA sequences of *AtNPR1* were cloned into the pGBKT7 vector in-frame with the GAL4 DNA-binding domain (BD). The cDNA sequences of target proteins were cloned into the pGADT7 vector in-frame with the GAL4 DNA-activating domain (AD). Co-transformation of BD and AD vectors in Y2HGold was performed to confirm the interaction. Empty BD and AD vectors were used as negative controls. Additionally, standard positive controls (pGBKT7-53 and pGADT7-T; Clontech) and standard negative controls (pGBKT7-Lam and pGADT7-T) were included. Yeast transformants were plated on double dropout medium (DDO) with -Trp and -Leu and were screened on quadruple dropout medium (QDO) with -Trp, -Leu, -Ade, and -His supplemented with X-α-Gal and aureobasidin A (QDO/X/A). QDO/X/A was supplemented with 100 µM SA (Sigma Aldrich, Saint Louis, MO, USA). 

## Figures and Tables

**Table 1:** Summary of sequencing data from the different replicates of the NPR1-2 transgenic line and control non-transgenic ‘Valencia’ line.

Sample *	Clean Reads	Clean Bases	≥Q30 (%)	GC Content (%)	Total Mapped (%)	Uniquely Mapped (%)
NPR1_1	62,607,264	7.91G	92.89	44.84	47,656,347 (76.12%)	46,101,606 (73.64%)
NPR1_2	59,175,150	7.40G	92.79	45.23	45,972,884 (77.69%)	44,572,333 (75.32%)
NPR1_3	59,225,298	7.41G	92.58	44.92	47,206,105 (79.71%)	45,624,108 (77.03%)
Val_1	65,551,728	8.20G	92.44	44.4	54,260,656 (82.78%)	52,711,952 (80.41%)
Val_2	65,372,880	8.18G	92.84	44.19	54,287,742 (83.04%)	52,522,887 (80.34%)
Val_3	61,717,406	7.72G	92.7	44.59	51,647,046 (83.68%)	50,005,354 (81.02%)
Total/Average	373,649,726	47.00G	92.71	44.69	50,171,797 (80.50%)	48,589,707 (77.96%)

* NPR1_1/2/3 represent three replicates of NPR1-2 line while Val_1/2/3 represent three replicates of the control non-transgenic ‘Valencia’ line.

**Table 2:** List of all DEGs between the NPR1-2 transgenic line and control non-transgenic ‘Valencia’ line.

Gene ID	log_2_ (Fold Change)	padj	Functional Description	E-Value
Cs9g18210	Inf	2.76E-04	Cyclic nucleotide-gated ion channel 1	1.26E-100
orange1.1t01447	10.70	2.35E-03	Transactivator/viroplasmin protein	2.95E-06
orange1.1t01448	10.36	3.52E-04	Putative Polyprotein CP	2.51E-07
Cs9g18180	9.60	2.48E-32	Cyclic nucleotide-gated ion channel 1	2.41E-98
orange1.1t04318	6.71	1.70E-19	Ankyrin repeat-containing protein	1.39E-15
orange1.1t04316	6.52	2.20E-19	Ankyrin repeat-containing protein	5.38E-21
Cs1g13910	6.34	1.76E-02	Wall-associated receptor kinase-like 10	3.06E-167
Cs9g18240	5.91	3.92E-02	Cyclic nucleotide-gated ion channel 1	1.81E-129
Cs1g03870	5.27	5.92E-05	Probable WRKY transcription factor 51	8.66E-38
orange1.1t04319	5.26	8.15E-05	Ankyrin repeat-containing protein	5.68E-07
Cs2g27430	4.00	2.22E-03	MYB-related protein Myb4	1.25E-50
orange1.1t04443	3.97	3.98E-03	TMV resistance protein N	1.09E-39
orange1.1t03802	3.96	3.80E-02	Protein Enhanced disease susceptibility 1	3.56E-135
Cs2g27410	3.86	4.63E-05	MYB-related protein Myb4	8.93E-51
orange1.1t04313	3.85	2.11E-05	Huamn Ankyrin-2	1.79E-15
Cs2g01090	3.81	1.23E-04	UPF0481 protein	8.09E-36
Cs7g11940	3.77	1.13E-02	Cytosolic sulfotransferase 12	4.84E-116
Cs1g24440	3.62	2.76E-04	Salicylate carboxymethyl transferase	6.62E-158
orange1.1t03083	3.33	3.98E-03	Putative ribonuclease H protein	3.42E-20
Cs1g11960	3.31	1.15E-04	G-type lectin S-receptor-like serine/threonine-protein kinase	6.12-131
Cs5g16310	3.17	4.40E-05	Flavanone 3-dioxygenase	2.30E-69
orange1.1t02071	3.13	8.55E-05	UPF0481 protein	2.10E-28
Cs2g21000	3.04	3.17E-04	Linolenate hydroperoxide lyase, chloroplastic	0
Cs2g13280	3.03	8.73E-05	Probable receptor-like protein kinase	5.18E-98
orange1.1t04913	3.02	2.44E-03	TMV resistance protein N	1.45E-54
Cs7g29570	3.00	8.73E-05	Probable WRKY transcription factor 70	1.61E-41
Cs4g04210	2.84	2.92E-04	Transcription factor bHLH35	4.47E-67
orange1.1t01840	2.83	1.05E-02	TMV resistance protein N	8.85E-172
Cs4g05900	2.72	1.06E-02	Ankyrin repeat-containing protein	3.72E-27
Cs3g10110	2.62	2.54E-03	LRR receptor-like serine/threonine-protein kinase	9.01E-17
orange1.1t02504	2.55	3.90E-03	Probable disease resistance protein	3.26E-175
Cs4g18320	2.49	4.46E-03	Glutaredoxin-C6	2.03E-57
Cs9g12160	2.40	6.88E-03	LRR receptor-like serine/threonine-protein kinase GSO1	3.67E-14
Cs7g29040	2.34	2.61E-02	Heat shock cognate 70 kDa protein	0
Cs7g15430	2.31	1.25E-02	Cytochrome P450 89A2	3.06E-171
Cs5g04250	2.25	2.55E-02	Mitochondrial outer membrane protein porin of 36 kDa	3.27E-51
Cs6g01870	2.18	2.61E-02	Probable LRR receptor-like serine/threonine-protein kinase	6.63E-13
orange1.1t00589	2.14	3.59E-02	NAC domain-containing protein 68	1.50E-20
Cs5g06000	2.13	3.10E-02	Copper methylamine oxidase	3.84E-167
Cs1g03700	2.09	3.15E-02	Mechanosensitive ion channel protein 10	1.20E-13
Cs5g12280	−6.39	6.99E-05	Agamous-like MADS-box protein AGL8	3.38E-33
Cs5g12290	−5.76	1.70E-06	Agamous-like MADS-box protein AGL8	9.58E-34
Cs7g11800	−3.65	8.18E-06	Developmental protein SEPALLATA 2	8.38E-33
Cs9g07970	−2.33	8.25E-03	3-ketoacyl-CoA synthase 11	0
orange1.1t04139	−2.29	1.01E-02	Geraniol 8-hydroxylase	0

## Supplementary Materials

Supplementary materials can be found at https://www.mdpi.com/1422-0067/21/6/2009/s1.
