# Supplementary material for: Potential Mechanisms of AtNPR1 Mediated Resistance against Huanglongbing (HLB) in Citrus
Source: Int J Mol Sci. 2020 Mar 16;21(6):2009. doi: 10.3390/ijms21062009 (PMC7139736; doi:10.3390/ijms21062009)
Supplement: Supplementary file 1 [file ijms-21-02009-s001.pdf]

Supplementary Information

Supplementary Table S1: Primers of 12 DEG selected for qRT-PCR analysis and the *β-actin* housekeeping gene.

Accession No.	Primer sequence（5'-3'）	
	Forward	Reverse
Cs3g17260	GACAAAGGGTGACCGGATTAT	GGCCACCTTCTGCTTTAGAT
Cs9g18180	ATTTCGGAGGGAGAGTCAATTC	TCCTGACGTTTCAGTCCAATC
Cs7g29040	TCGGTTCAGGAAGACATCAAG	GCAAGCACCATTGAAGAGATG
Cs1g23200	TCGGGCATCAACATGAAGAG	TTCTTCGGCAGGAAGTGATG
Cs8g10250	CTCCATTCGCAGAACCTTCA	TCTTAGTTCACGGGTCCTATCT
Cs3g15890	CACTCTGTTTCTCGCCAAATTC	GCCTCAAACAACAACGACTG
Cs3g10110	CTTTAGACGGTAGCATTCCCTC	GAAAATCCAAGTAGACGCAACC
Cs1g13910	GCCACCGATTACTTCAACTTG	TTTCCATCTGCTAACATTCCTTTG
Cs7g03940	TCCAAGTACGCATAAAACCCC	GTTGGAGCGCACAATGTTATC
orange1.1t03391	GGTAAGGCCTTGCTCAATCT	GAGTTGAAGCACCATTGTTTAGG
orange1.1t02504	TTTCTCTTCCTAATTCCCTCGC	AAAACCTGACCAACATTGCAC
orange1.1t04319	CATTTGCCAGGAAGCTAAACC	CGAACAAGGTCCGGATCAATA
β-actin	GCTGCCTGATGGCCAGATC	AGTTGTAGGTAGTCTCATGAA

Supplementary Table S2: Accession numbers of genes validated through qRT-PCR

Common Name	Accession No. from the Citrus sinenesis annotation project (http://citrus.hzau.edu.cn/orange/)	Accession No. from the phytozome database https://phytozome.jgi.doe.gov/	Accession No. from the NCBI database
Cyclic nucleotide-gated ion channel	Cs9g18180	orange1.1g008614m	XP_024958326.1
LOB domain-containing protein 39	Cs3g15890	orange1.1g029809m	XM_006433561.2
Receptor-like protein 13 (LRR1)	Cs3g10110	orange1.1g042884m	XM_025096610.1
Heat shock cognate 70 kDa protein 2 (HSP70-2)	Cs7g29040	orange1.1g005824m	XM_025101446.1
Cullin-3A	Cs7g03940	orange1.1g007158m	XM_006483113.2
MADS-box protein SVP (SVP1)	Cs8g10250	orange1.1g040972m	XM_006472407.3
MADS-box protein SVP (SVP2)	Cs3g17260	orange1.1g040972m	XM_006472408.3
Methylesterase 1-related (SABP2)	Cs1g23200	orange1.1g024065m	XM_006466598.3
Wall-associated receptor kinase-like 10	Cs1g13910	orange1.1g044312m	XM_006465368.3
Leucine-rich repeat-containing protein	orange1.1t02504	orange1.1g042541m	XM_006441973.2

Agamous-like MADS-box protein AGL80	orange1.1t03391	orange1.1g040642m	XM_006478167.2
Ankryin-domain protein	orange1.1t04319	orange1.1g043149m	XM_025099106.1
β-actin	Cs1g05000	orange1.1g017124m	XM_006464503.3

Supplementary Table S3: Primers used for gene cloning

Purpose	Primer name	Primer sequence（5'-3'）
attB-PCR for Gateway	AtNPR1-gF	GGGGACAAGTTTGTACAAAAAAGCAGGCTCCATGGACACCACCATTGATGG
	AtNPR1-gR	GGGGACCACTTTGTACAAGAAAGCTGGGTCTCACCGACGACGATGAGAGAG
	AtNPR3-gF	GGGGACAAGTTTGTACAAAAAAGCAGGCTCCATGGCTACTTTGACTGAGC
	AtNPR3-gR	GGGGACCACTTTGTACAAGAAAGCTGGGTCTCATGTTGTGTTGTGCAGG
	CsNPR3-gF	GGGGACAAGTTTGTACAAAAAAGCAGGCTCCATGGCTAATTCGATTGAAC
	CsNPR3-gR	GGGGACCACTTTGTACAAGAAAGCTGGGTCTCATAATTTTCCGAGCTTGT
	CsTGA2-gF	GGGGACAAGTTTGTACAAAAAAGCAGGCTCCATGCCGAGCTTTGATTCTC
	CsTGA2-gR	GGGGACCACTTTGTACAAGAAAGCTGGGTCTCACTCTCTTGGGCGGGC
	CsTGA5-gF	GGGGACAAGTTTGTACAAAAAAGCAGGCTCCATGGAGAATGCTGTTGACC
	CsTGA5-gR	GGGGACCACTTTGTACAAGAAAGCTGGGTATCACTCCCGTGGCCGGGC
